# Asthma and allergy prevalence among children at risk for developmental coordination disorder

**DOI:** 10.1111/pai.70370

**Published:** 2026-05-15

**Authors:** Erin Pitt, Kathryn Fortnum, Matthew Bourke, John Cairney, Jennifer Koplin

**Affiliations:** ^1^ Child Health Research Centre The University of Queensland Brisbane Queensland Australia; ^2^ Health and Wellbeing Centre for Research Innovation, School of Human Movement and Nutrition Sciences The University of Queensland Brisbane Queensland Australia; ^3^ Centre for Food Allergy Research Murdoch Children's Research Institute Melbourne Victoria Australia; ^4^ Population Allergy Group Murdoch Children's Research Institute Melbourne Victoria Australia

**Keywords:** allergy, asthma, children, developmental coordination disorder, neurodevelopmental disorders

To the Editor,

There is emerging evidence to suggest an association between allergic disease and neurodevelopmental disorders.^1^, ^2^ Recent studies have reported a possible link between self‐reported asthma or food allergies and neurodevelopmental disorders^1^ and gross motor delay,^3^ however specific neurodevelopmental disorders were not examined.

Developmental Coordination Disorder (DCD) is a neurodevelopmental condition characterized by difficulties with coordination, including fine and gross motor skill issues, subsequently affecting daily activities and learning.^4^ A life‐long condition, DCD often co‐occurs with other childhood disorders and conditions such as attention deficit hyperactivity disorder (ADHD) or autism spectrum disorder (ASD).^5^ It is not known whether children at risk of DCD, distinct from neurodevelopmental conditions more broadly, have an increased likelihood of developing asthma or allergies compared to their neurotypically developing peers. The aim of this study was to explore whether children at risk of DCD were more likely to develop parent‐reported asthma or allergies compared to their neurotypically developing peers.

This analysis used data from The *Coordination and Activity Tracking in CHildren (CATCH) study*, a Canadian prospective cohort study investigating the complex relationship between DCD, physical activity, physical fitness and body composition from early to mid‐childhood. The cohort aimed to recruit a sample of 300 children at risk of DCD and 300 children with typical motor development.^6^ Commencing in 2013, CATCH recruited children aged 4–5 years up until 2017 who have been followed up annually for 4 years. Details of recruitment methods, data collection and cohort profile for the CATCH study have been reported previously.^6^, ^7^ Additional methods supporting the current analysis can be found in Appendix S1.

Potential cases of DCD were identified through implementation of the Movement Assessment Battery for Children 2nd Edition (MABC‐2).^8^ Raw scores on the MABC‐2 were converted into a standard score and percentiles in accordance with the age of the child. A single measure of motor proficiency was retrospectively calculated for each child as an average of MABC‐2 scores across years 1–4, providing two or more datapoints were available. The benefits of this approach include reducing measurement error and accounting for natural progression in motor skills.^7^ A three‐level categorical variable of motor proficiency was used for this analysis; typically developing (TD; >16th percentile), at risk for DCD (DCDr; 6th–16th percentile), and probable DCD (pDCD; <6th percentile). The primary outcomes of interest were cumulative parent‐reported presence of any allergy or asthma in the children from 4–5 to 8–9 years of age, defined as any instance of parent‐reported allergy/asthma over the four time points.

Descriptive statistics are presented as frequencies, mean and standard deviation or median and inter‐quartile range, depending on the distribution of the data. Chi‐Squared tests were used to investigate group differences for categorical variables as well as parametric and non‐parametric *t*‐tests and ANOVA, depending on the distribution of the data. Logistic regression was undertaken to model the association between DCD and asthma or allergy. Clinical and demographic characteristics of the cohort selected a priori as potential confounders included presence of ADHD and ASD (collected using the ADHD index of the Conners' Parent Rating Scales and Child Behavioral Checklist (CBCL), respectively), family history of neurodevelopmental conditions, primary caregiver age, highest level of education and ethnicity, socio‐economic status, season of birth and duration of breastfeeding.^6^, ^7^ Models also incorporated ADHD and ASD as interaction terms to investigate their conditional effect with DCD status on asthma and allergy.

A total of 589 children were recruited to the CATCH study. A small portion of participants did not have the minimum two data points required to establish their motor proficiency status and were thus excluded from this analysis (*n* = 42, 7.1%), resulting in a final sample of 547. Probable DCD was identified in 13.9% of children and a further quarter (24.0%) were classified as DCDr. The majority of the cohort were Caucasian (85.6%), more than half were male (57.4%), and just under two thirds of primary carers had a university qualification as their highest level of education (64.3%). The prevalence of ASD and ADHD was 6.4% and 15.2% respectively, and almost one in ten had a family history of neurodevelopmental disorders (9.3%); all of which were significantly higher in children with pDCD and DCRr (Table 1).

**TABLE 1:** Characteristics of the sample stratified by DCD risk status.

Characteristics	Total Sample N (%) *N* = 547	DCD status			*p*‐value
		TD *N* = 340 (62.2%)	DCDr *N* = 131 (24.0%)	pDCD *N* = 76 (13.9%)	
Any report of asthma					
No	460 (84.1)	289 (85.0)	108 (83.2)	62 (81.6)	.724
Yes	87 (15.9)	51 (15.0)	22 (16.8)	14 (18.4)	
Any report of allergy					
No	394 (72.0)	255 (74.7)	90 (68.7)	50 (65.8)	.159
Yes	153 (28.0)	85 (25.3)	41 (31.3)	26 (34.2)	
Sex					
Male	314 (57.4)	170 (50.0)	85 (64.9)	59 (77.6)	<.001
Female	233 (42.6)	170 (50.0)	46 (35.1)	17 (22.4)	
Siblings					
No	377 (68.9)	242 (71.2)	84 (64.1)	51 (67.1)	.311
Yes	170 (31.1)	98 (28.8)	47 (35.9)	25 (32.9)	
Primary caregiver's age at birth of child (Median; IQR) (missing = 11)	37.0; 34.0–40.0	37.0; 34.5–40.5	37.0; 34.0–40.0	35.5; 33.0–38.0	.022
Primary carer's highest level of education (missing *n* = 6)					
High school or less	64 (11.8)	36 (10.7)	17 (13.0)	11 (15.1)	.165
College or technical training	129 (23.8)	71 (21.1)	36 (27.5)	22 (30.1)	
University	348 (64.3)	230 (68.3)	78 (59.5)	40 (54.8)	
Primary carer's ethnicity (missing *n* = 6)					
Caucasian	463 (85.6)	287 (86.2)	107 (82.3)	69 (93.2)	.303
East Asian	18 (3.3)	11 (3.3)	6 (4.6)	1 (1.4)	
Other	60 (11.1)	39 (11.6)	17 (13.1)	4 (5.4)	
ADHD (missing *n* = 9)					
No	456 (84.8)	299 (89.3)	102 (80.3)	55 (72.4)	<.001
Yes	82 (15.2)	36 (10.8)	25 (19.7)	21 (27.6)	
ASD					
No	512 (93.6)	327 (96.2)	120 (91.6)	65 (85.5)	.002
Yes	35 (6.4)	13 (3.8)	11 (8.4)	11 (14.5)	
Family history of neurodevelopmental disorders					
No	496 (90.7)	318 (93.5)	113 (86.3)	65 (85.5)	.013
Yes	51 (9.3)	22 (6.5)	18 (13.7)	11 (14.5)	
Season of birth					
Winter	146 (26.7)	89 (26.2)	37 (28.2)	20 (26.3)	.449
Spring	136 (24.9)	79 (23.2)	37 (28.2)	20 (26.3)	
Summer	140 (25.6)	85 (25.0)	31 (23.7)	24 (31.6)	
Autumn	125 (22.9)	87 (25.6)	26 (19.9)	12 (15.8)	
Duration of breastfeeding (missing *n* = 15)					
None	16 (3.0)	7 (2.1)	5 (3.9)	4 (5.4)	.184
Up to 6 months	134 (25.2)	79 (23.9)	31 (24.4)	24 (32.4)	
6 months or more	382 (71.8)	245 (74.0)	91 (71.7)	46 (62.2)	

*Note*: Percentages may not add to 100 due to rounding.

Abbreviations: ADHD, Attention Deficit Hyperactivity Disorder; ASD, Autism Spectrum Disorder; DCD, Developmental Coordination Disorder; IQR, Inter Quartile Range; TD, Typically Developing; DCDr, at risk for Developmental Coordination Disorder; pDCD, probable Developmental Coordination Disorder.

The prevalence of asthma and allergy within the cohort was 15.9% and 28.0%, respectively (Table 1). There was no statistically significant association between DCD and risk of developing asthma or allergy. The prevalence of asthma and allergy was higher among males and among children with primary caregivers who were younger (Tables 2 and 3). Children with a family history of neurodevelopmental disorders were 2.2 times more likely to have allergy compared to those without a family history of neurodevelopmental disorders (95% CI 1.14–4.24, *p* = .019) (Tables 2 and 3). ADHD was associated with asthma and allergy in unadjusted analyses (Table 2) but not in adjusted analyses (Table 3).

**TABLE 2:** Characteristics of the sample stratified by presence of asthma and allergy.

Characteristic	*N* (%) asthma	*p*‐Value	*N* (%) allergy	*p*‐Value
DCD Status				
TD	51 (15.0)	.724	85 (25.0)	.159
DCDr	22 (16.8)		41 (31.3)	
pDCD	14 (18.4)		26 (34.2)	
Sex				
Male	61 (19.4)	.009	101 (32.2)	.008
Female	26 (11.2)		51 (21.9)	
Siblings				
No	57 (15.1)	.454	114 (30.2)	.057
Yes	30 (17.7)		38 (22.4)	
Primary carer's age at birth of child (Median; IQR) (missing = 11)	38; 34.0–41.0	.311	38.0; 35.0–41.0	.037
Primary carer's highest level of education (missing *n* = 6)				
High school or less	10 (15.6)	.092	18 (28.1)	.924
College or technical training	28 (21.7)		34 (26.4)	
University	47 (13.5)		98 (28.2)	
Primary carer's ethnicity (missing *n* = 6)				
Caucasian	75 (16.4)	.676	127 (27.4)	.989
East Asian	2 (11.1)		5 (27.8)	
Other	7 (11.7)		17 (28.3)	
ADHD (missing *n* = 9)				
No	63 (13.8)	.003	120 (26.3)	.056
Yes	22 (26.8)		30 (36.6)	
ASD				
No	78 (15.2)	.101	140 (27.3)	.375
Yes	9 (25.7)		12 (34.3)	
Family history of neurodevelopmental disorders				
No	77 (15.5)	.448	129 (26.0)	.004
Yes	10 (19.6)		23 (45.1)	
Season of birth				
Winter	22 (15.1)	.431	50 (34.3)	.180
Spring	21 (15.4)		38 (27.9)	
Summer	28 (20.0)		35 (25.0)	
Autumn	16 (12.8)		29 (23.2)	
Duration of breastfeeding				
None	5 (31.3)	<.001	8 (50.0)	.055
Up to 6 months	34 (25.4)		41 (30.6)	
6 months or more	44 (11.5)		96 (25.1)	

*Note*: Percentages may not add to 100 due to rounding.

Abbreviations: ADHD, Attention Deficit Hyperactivity Disorder; ASD, Autism Spectrum Disorder; DCD, Developmental Coordination Disorder; IQR, Inter Quartile Range; TD, Typically Developing; DCDr, at risk for Developmental Coordination Disorder; pDCD, probable Developmental Coordination Disorder.

**TABLE 3:** Predictors of asthma and allergy prevalence.

Variable	Asthma		Allergy	
	aOR^a^	*p*‐value	aOR^a^	*p*‐Value
DCD status				
TD	1.0	‐	1.0	‐
DCDr	0.78 (0.39–1.57)	.491	1.50 (0.89–2.53)	.124
pDCD	0.72 (0.29–1.77)	.478	0.96 (0.46–1.97)	.900
Child Sex				
Female	1.0	‐	1.0	‐
Male	1.88 (1.09–3.25)	.023	1.64 (1.07–2.52)	.023
Family history of neurodevelopmental disorders				
No	1.0	‐	1.0	‐
Yes	1.06 (0.47–2.38)	.888	2.20 (1.14–4.24)	.019
Primary carer's highest level of education				
High school or less	1.0	‐	1.0	‐
College or technical	1.17 (0.50–2.71)	.722	0.91 (0.44–1.88)	.798
University	0.69 (0.32–1.50)	.346	0.92 (0.48–1.76)	.792
Primary carer's ethnicity				
Caucasian	1.0	‐	1.0	‐
East Asian	0.66 (0.14–3.17)	.605	0.69 (0.21–2.27)	.544
Other	0.85 (0.36–2.01)	.713	1.23 (0.66–2.31)	.517
Primary caregiver's age at birth of child	1.05 (1.00–1.10)	.034	1.05 (1.01–1.09)	.019
Siblings				
No	1.0	‐	1.0	‐
Yes	0.95 (0.56–1.63)	0.869	0.66 (0.42–1.05)	.078
Season of birth				
Winter	1.0	‐	1.0	‐
Spring	1.09 (0.55–2.16)	.815	0.89 (0.52–1.52)	.670
Summer	1.23 (0.63–2.40)	.542	0.67 (0.38–1.15)	.147
Autumn	0.94 (0.45–1.95)	.866	0.57 (0.32–1.03)	.062
Presence of ADHD				
No	1.0	‐	1.0	‐
Yes	1.06 (0.39–2.90)	.904	1.60 (0.72–3.59)	.251
ADHD and DCD status interaction				
TD × No ADHD	1.0	‐	1.0	‐
DCDr × ADHD	2.11 (0.48–9.25)	.320	0.36 (0.09–1.36)	.132
pDCD × ADHD	2.21 (0.38–12.77)	.375	1.32 (0.30–5.78)	.717
Presence of ASD				
No	1.0	‐	1.0	‐
Yes	0.55 (0.06–4.66)	.579	1.03 (0.25–4.21)	.973
ASD and DCD status interaction				
TD × No ASD	1.0	‐	1.0	‐
DCDr × ASD	2.85 (0.20–39.79)	.437	0.59 (0.07–5.18)	.635
pDCD × ASD	2.60 (0.16–42.41)	.503	1.81 (0.21–15.33)	.588

Abbreviations: ADHD, Attention Deficit Hyperactivity Disorder; ASD, Autism Spectrum Disorder; DCD, Developmental Coordination Disorder; TD, Typically Developing; DCDr, at risk for Developmental Coordination Disorder; OR, Odds Ratio; pDCD, probable Developmental Coordination Disorder.

^a^ Models adjusted for ADHD and ASD, family history of neurodevelopmental conditions, primary caregiver age, highest level of education and ethnicity, socio‐economic status, season of birth and duration of breastfeeding.

To our knowledge, this is the first study to estimate the prevalence of asthma and allergy among children with pDCD, building on existing research exploring allergy among children with neurodevelopmental conditions more broadly.^1^ We found no significant differences between motor proficiency groups and the likelihood of developing parent‐reported asthma or allergy, despite some evidence of an association between ADHD and both asthma and allergy in unadjusted analyses. Although asthma and allergy outcomes in this cohort relied on parent report, previous studies reporting a link between other developmental disorders and allergic outcomes have also used self‐ or parent report, thus it is possible that different types of developmental disorders have different associations with these outcomes.

A strength of this study is the well‐characterized DCD outcomes, which differ from previous studies that included broad, non‐specific definitions of developmental disorders. There are several limitations. Firstly, it was not possible to accurately distinguish between a new or existing allergy via the self‐reported questionnaire responses, thus incidence of asthma and allergy at different ages could not be established, and only cumulative prevalence is reported. Further, both asthma and allergy were self‐reported, thus misclassification is possible, and information on the type of allergy (e.g. food vs. respiratory allergy) was not available. Screening tools used to determine the presence of ADHD and/or ASD may not have accurately captured their prevalence, thus data around the association between ADHD and ASD and allergies should be interpreted with caution.

Data on family history of allergic conditions was not collected as part of the CATCH study, limiting the ability to explore its effect in the current analysis. It is possible the current analysis was not sufficiently powered to detect associations between DCD status and allergy or asthma, in which future studies with larger sample sizes are warranted to further investigate this relationship. Finally, DCD is a heterogeneous condition, with several subtypes of DCD with varying levels of impairment in gross and fine motor being observed.^9^ These heterogenous subtypes of DCDE, which could influence the association with allergies and asthma, were not examined in the current analysis.

It is vital to address the long‐term consequences of neurodevelopmental conditions in children, including DCD, and improve the identification and management of co‐occurring conditions. Whilst no association was evident between DCD and asthma or allergy prevalence in this cohort, evidence for the co‐occurrence of allergic outcomes and other neurodevelopmental conditions is growing.^10^, ^11^, ^12^ Continued investigation into these associations may support earlier diagnosis and more effective management, thereby improving quality of life outcomes for children with neurodevelopmental conditions.

## AUTHOR CONTRIBUTIONS

**Erin Pitt:** Conceptualization; investigation; methodology; visualization; formal analysis; data curation; project administration; writing – original draft; writing – review and editing. **Jennifer Koplin:** Conceptualization; methodology; writing – review and editing; supervision. **Kathryn Fortnum:** Conceptualization; methodology; visualization; resources; writing – review and editing. **Matthew Bourke:** Conceptualization; methodology; visualization; resources; writing – review and editing. **John Cairney:** Conceptualization; methodology; funding acquisition; supervision; writing – review and editing.

## FUNDING INFORMATION

The CATCH study is funded by the Canadian Institute of Health Research (CIHR Award #: MOP 126015). KF is funded by the Health and Wellbeing Centre for Research Innovation (HWCRI), which is co‐funded by the University of Queensland and Health and Wellbeing Queensland. MB, JK, and EP are supported by the University of Queensland Strategic Funding for the 360‐Kids Community Network Health Research Accelerator (HERA) program.

## CONFLICT OF INTEREST STATEMENT

The authors have no conflicts of interest to declare.

## ETHICS STATEMENT

The study was approved by McMaster University (Hamilton Integrated Research Ethics Board #15975). All participants provided written informed consent.

## Supporting information


Appendix S1.

## Data Availability

The data that support the findings of this study are available from the corresponding author upon reasonable request.
