# Supplementary material for: Asthma and allergy prevalence among children at risk for developmental coordination disorder
Source: Pediatr Allergy Immunol. 2026 May 15;37:e70370. doi: 10.1111/pai.70370 (PMC13176891; doi:10.1111/pai.70370)
Supplement: Supplementary file 1 — Appendix S1. [file PAI-37-e70370-s001.docx]

**Supplementary Methods**

1. **Data collection methods:**

At baseline, eligible children completed Phase 1 of baseline testing which included assessments of cognitive ability (IQ), physical fitness and body composition. The Movement Assessment Battery for Children 2^nd^ Edition (MABC-2)^1^ was used to identify potential cases of DCD. Parents and children completed an array of questionnaires and a semi-structured interview was also completed by parents. The questionnaires collected information on family demographics, medical conditions (including asthma and allergy), assessment of behavioural and emotional problems, measures of health and risk factors for obesity, and participation in physical activity.

Children who scored at or below the 16^th^ percentile for MABC-2 and a random sample of those scoring above 16^th^ percentile were invited into the longitudinal phase of the study and continue testing. Phase 2 of baseline testing included additional measures of body composition, physical functioning, motor skills and physical activity. All baseline assessments were repeated once per year for 4 years, except IQ and assessment for ADHD (collected twice during years 2-4), resulting in 4 waves of data collection.

1. **Outcomes and Exposures**

**2.1 Primary outcomes:**

Data on allergy and asthma occurrence was obtained via parent self-report using questionnaires at each of the four time points which asked whether the child participant had been diagnosed with any medical conditions (yes/no), of which two of the standardised options were allergy and asthma. This was followed by an open-ended question to detail specific information about the condition diagnosed. The cumulative prevalence of allergy or asthma in the cohort was taken to be any instance of self-report over the four time points, regardless of whether it was a new or existing occurrence. This measure did not take into account children who may have outgrown their allergy over the duration of the study as this information was not captured.

**2.2 Exposure:**

Motor proficiency and subsequently potential DCD was measured prospectively at the four time points using the Movement Assessment Battery for Children 2^nd^ Edition (MABC-2).^1^ This is a standardized motor coordination assessment tool consisting of most eight motor tasks widely used for the identification of DCD.^1^ It has been shown to be a valid and reliable tool for the assessment of movement difficulties, including in children as young as 3-6 years of age. Raw scores on the MABC-2 are converted into a standard score and percentiles in accordance with the age of the child. The average score was taken from the repeated assessments. The categorization was based on this average, not from categorizations at each individual assessment.

**2.3 Other measures:**

The ADHD index of the Conners’ Parent Rating Scales was completed twice by parents during years 2-4, covering diagnostic criteria of the DSM-IV.^2^ Presence of ADHD was categorised as a binary variable (yes/no). Children were identified as having ADHD if they met the criteria for ADHD during at least one of the two data collection time points, defined as test scores ≥6 (out of 9) on one or both subscales. Parents also completed the Child Behavioural Checklist (CBCL) used to assess behavioural, emotional and autism-like behaviours.^3^ Presence of Autism Spectrum Disorders (ASD) (yes/no) was defined based on t-score clinical cut off values during at least one data collection time point. Family history of neurodevelopmental conditions was a composite binary variable (yes/no) generated from individual variables of self-reported family history of ADHD (yes/no), ASD (yes/no) or DCD (yes/no) collected across all data collection time points.

**2.4 Data Analysis:**

As the cohort was recruited to answer research questions focussed on DCD, power calculations for the current research questions were not performed. We have not conducted any additional retrospective power calculations for the current manuscript due to potential limitations of retrospective power calculations^4^ and potential limitations of power calculations for additional analyses of existing data sets.^5^

Separate binary logistic regression models were estimated to quantify the association between DCD status and asthma and allergy. Clinical and demographic characteristics of the CATCH cohort were explored as potential confounders of the association between risk of DCD and asthma or allergy prevalence. Variables of interested included maternal age at birth of child and highest level of education, ethnicity, child sex, family history of neurodevelopmental disorders, season of birth, having siblings, presence of ADHD and presence of ASD.

Duration of breastfeeding was reported descriptively as a three-category variable (None; Up to 6 months; 6 months or more). Despite small numbers in the category where participants were not breastfed, we were still interested in reporting the differences in asthma and allergy prevalence across duration of breastfeeding. It is often an interesting and important predictor for a range of health outcomes including asthma and allergy, especially where participants do and do not receive breastmilk.^6^ Duration of breastfeeding has been reported descriptively, however was not a reliable variable to include in the logistic regression modelling. Similarly, Asian ethnicity is often associated with increased allergy prevalence^7^ and was considered important to explore in the current analysis, both descriptively and in the logistic regression.

A stepwise model building approach was taken in which each variable was added to the model separately with DCD status and asthma or allergy to discern if there was either a confounding effect (modification of the association between DCD status and asthma or allergy by ≥10%) or the presence of the variable approached statistical significance (p<0.2).^8^ Variables were then added one at a time to assess the effect on DCD status and the overall model. The final parsimonious model endeavoured to explain the most amount of variation with the fewest variables.^8^

**References:**

1. Henderson S, Sugden D, Barnett A. *Movement Assessment Battery for Children Examiner's Manuel (2nd ed.)*. London: Harcourt Assessment 2007.

2. Conners K. *Conners 3rd Edition*. Toronto: Mulit-Health Systems 2008.

3. Achenback T, Rescorla L. *Child behaviour checklist for ages 1½-5, Achenbach System of Emprically Based Assessment.* Burlington: University of Vermont 2000.

4. Zumbo BD, Hubley AM. A Note on Misconceptions Concerning Prospective and Retrospective Power. *Journal of the Royal Statistical Society Series D (The Statistician).* 1998;47:385-88. doi:10.1111/1467-9884.00139.

5. Hernan MA. Causal analyses of existing databases: no power calculations required. *Journal of clinical epidemiology.* 2022;144:203-05. doi:10.1016/j.jclinepi.2021.08.028.

6. Lodge CJ, Tan DJ, Lau MXZ, et al. Breastfeeding and asthma and allergies: a systematic review and meta-analysis. *ACTA PAEDIATRICA.* 2015;104:38-53. doi:10.1111/apa.13132.

7. Suaini NHA, Loo EXL, Peters RL, et al. Children of Asian ethnicity in Australia have higher risk of food allergy and early‐onset eczema than those in Singapore. *Allergy.* 2021;76:3171-82. doi:10.1111/all.14823.

8. Sahay A. *Applied regression and modeling: a computer integrated approach*. New York: Business Expert Press 2016.
